# 2-[(*E*)-(3-Carb­oxy-4-hy­droxy­phen­yl)iminiometh­yl]-4-chloro­phenolate

**DOI:** 10.1107/S1600536810033362

**Published:** 2010-08-28

**Authors:** Abeer Mohamed Farag, Teoh Siang Guan, Hasnah Osman, Madhukar Hemamalini, Hoong-Kun Fun

**Affiliations:** a School of Chemical Sciences, Universiti Sains Malaysia, 11800 USM, Penang, Malaysia; b X-ray Crystallography Unit, School of Physics, Universiti Sains Malaysia, 11800 USM, Penang, Malaysia

## Abstract

The title Schiff base compound, C_14_H_10_ClNO_4_, has been synthesized by the reaction of 5-amino-2-hy­droxy­benzoic acid and 5-chloro-2-hy­droxy­benzaldehyde. The mol­ecule is a zwitterion in the crystal, with the phenolic hy­droxy group deprotonated and the imine N atom protonated. It adopts an *E* configuration about the central C=N double bond. The dihedral angle between the two benzene rings is 3.83 (7)°. Intra­molecular N—H⋯O and O—H⋯O hydrogen bonding generates *S*(6) ring motifs. In the crystal, mol­ecules are connected by inter­molecular O—H⋯O and C—H⋯Cl hydrogen bonds, forming a supra­molecular chain.

## Related literature

For applications of Schiff bases, see: Youssef *et al.* (2009 ▶); Salih & Hamdi (2008 ▶); Belaid *et al.* (2006 ▶); Karthikeyan *et al.* (2006 ▶). For hydrogen-bond motifs, see: Bernstein *et al.* (1995 ▶). For the stability of the temperature controller used in the data collection, see: Cosier & Glazer (1986 ▶).
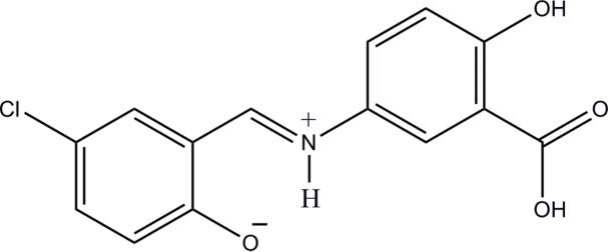


## Experimental

### 

#### Crystal data

C_14_H_10_ClNO_4_
*M* *_r_* = 291.68
Monoclinic, 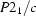

*a* = 7.1504 (6) Å
*b* = 10.9059 (10) Å
*c* = 15.8015 (18) Å
β = 98.396 (2)°
*V* = 1219.0 (2) Å^3^
*Z* = 4
Mo *K*α radiation
μ = 0.33 mm^−1^
*T* = 100 K
0.36 × 0.08 × 0.05 mm

#### Data collection

Bruker APEXII DUO CCD area-detector diffractometer
Absorption correction: multi-scan (*SADABS*; Bruker, 2009 ▶) *T*_min_ = 0.892, *T*_max_ = 0.984
24711 measured reflections
3548 independent reflections
2839 reflections with *I* > 2σ(*I*)
*R*_int_ = 0.048

#### Refinement

*R*[*F* ^2^ > 2σ(*F* ^2^)] = 0.038
*wR*(*F* ^2^) = 0.111
*S* = 1.03
3548 reflections
189 parameters
H atoms treated by a mixture of independent and constrained refinement
Δρ_max_ = 0.33 e Å^−3^
Δρ_min_ = −0.26 e Å^−3^

### 

Data collection: *APEX2* (Bruker, 2009 ▶); cell refinement: *SAINT* (Bruker, 2009 ▶); data reduction: *SAINT*; program(s) used to solve structure: *SHELXTL* (Sheldrick, 2008 ▶); program(s) used to refine structure: *SHELXTL*; molecular graphics: *SHELXTL*; software used to prepare material for publication: *SHELXTL* and *PLATON* (Spek, 2009 ▶).

## Supplementary Material

Crystal structure: contains datablocks global, I. DOI: 10.1107/S1600536810033362/fj2328sup1.cif

Structure factors: contains datablocks I. DOI: 10.1107/S1600536810033362/fj2328Isup2.hkl

Additional supplementary materials:  crystallographic information; 3D view; checkCIF report

## Figures and Tables

**Table 1:** Hydrogen-bond geometry (Å, °)

*D*—H⋯*A*	*D*—H	H⋯*A*	*D*⋯*A*	*D*—H⋯*A*
O3—H1*O*3⋯O4^i^	0.97	1.56	2.5220 (16)	173
N1—H1*N*1⋯O4	0.85 (2)	1.78 (2)	2.5217 (17)	144.2 (19)
O1—H1*O*1⋯O2	0.96 (2)	1.67 (2)	2.5901 (17)	158 (2)
C7—H7*A*⋯Cl1^ii^	0.93	2.81	3.6603 (15)	152

Symmetry codes: (i) 
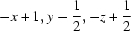
; (ii) 
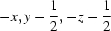
.

**Fig. 1.**
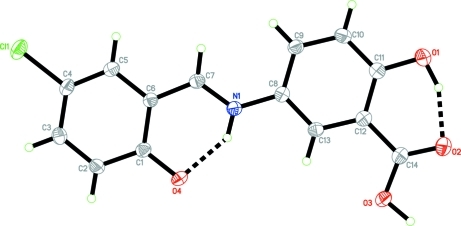
The asymmetric unit of the title compound. Displacement ellipsoids are drawn at the 50% probability level. Intramolecular interactions are shown as dashed lines.

**Fig. 2.**
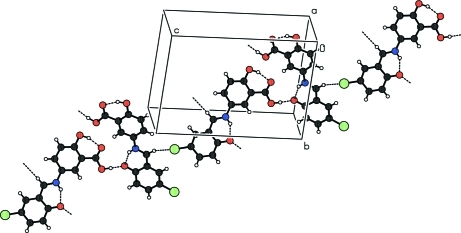
One-dimensional molecular chain generated by O—H···O and C—H···Cl hydrogen bonds.

## supplementary crystallographic information

### Comment

Schiff bases have received much attention, mainly because of their extensive application in the field of synthesis and catalysis (Youssef *et al.*, 2009; Salih & Hamdi, 2008). Schiff bases derived from ortho-phenylenediamine are of particular interests because of the proximity of the nitrogen atoms, which permits their simultaneous coordination to the same metal cation, leading to more stable compounds (Belaid *et al.*, 2006). Schiff base ligands are an important class of compounds, possessing a wide spectrum of biological and pharmacological activities such as antibacterial and antifungal (Karthikeyan *et al.*, 2006) properties.  Keeping in view of the importance of the Schiff bases, the title compound (I) was synthesized.

The molecule of (I), (Fig. 1), crystallizes in a zwitterionic form with cationic iminium and anionic phenolate i.e. the phenol -OH group was deprotonated and the imine N atom was protonated. (I) exists in a trans configuration about the C7═N1 bond [1.3061 (19) Å] with the torsion angle C6-C7-N1-C8 = 178.05 (13)°.  The dihedral angle between the two phenyl (C1–C6)/(C8–C13) rings is 3.83 (7)°.

In the crystal structure (Fig. 2), intramolecular N1—H1N1···O4 and O1—H1O1···O2 hydrogen bonding generates an *S*(6) ring motifs (Bernstein *et al.*, 1995). The crystal structure is further stabilized by intermolecular O3—H1O3···O4 and C7—H7A···Cl1 (Table 1) hydrogen bonds, to form one-dimensional chains.

### Experimental

To a stirred solution of 5-amino -2- hydroxybenzoic acid (0.40 g, 2.9 mmol) in  methanol  was added 5-chloro-2-hydroxybenzaldehyde (0.40 g, 2.5 mmol). The reaction was refluxed for 1 h at 70°C after which the precipitate formed was filtered and recrystallized from dichloromethane and methanol (1:1). Orange needle-shaped single crystals suitable for X-ray structure determination were formed after slow evaporation of solvent at room temperature.

### Refinement

Atoms H1N1 and  H1O1 were located from a difference Fourier map and were refined freely [N–H= 0.85 (2) Å and O–H= 0.96 (3) Å]. The remaining hydrogen atoms were positioned geometrically [C–H = 0.93 Å] and were refined using a riding model, with *U*_iso_(H) = 1.2 or 1.5 *U*_eq_(C, O).

### Crystal data

**Table:**

C_14_H_10_ClNO_4_	*F*(000) = 600
*M**_r_* = 291.68	*D*_x_ = 1.589 Mg m^−^^3^
Monoclinic, *P*2_1_/*c*	Mo *K*α radiation, λ = 0.71073 Å
Hall symbol: -P 2ybc	Cell parameters from 5212 reflections
*a* = 7.1504 (6) Å	θ = 2.9–29.8°
*b* = 10.9059 (10) Å	µ = 0.33 mm^−^^1^
*c* = 15.8015 (18) Å	*T* = 100 K
β = 98.396 (2)°	Needle, orange
*V* = 1219.0 (2) Å^3^	0.36 × 0.08 × 0.05 mm
*Z* = 4	

### Data collection

**Table:**

Bruker APEXII DUO CCD area-detector diffractometer	3548 independent reflections
Radiation source: fine-focus sealed tube	2839 reflections with *I* > 2σ(*I*)
graphite	*R*_int_ = 0.048
φ and ω scans	θ_max_ = 30.0°, θ_min_ = 2.3°
Absorption correction: multi-scan (*SADABS*; Bruker, 2009)	*h* = −10→10
*T*_min_ = 0.892, *T*_max_ = 0.984	*k* = −15→15
24711 measured reflections	*l* = −21→22

### Refinement

**Table:**

Refinement on *F*^2^	Primary atom site location: structure-invariant direct methods
Least-squares matrix: full	Secondary atom site location: difference Fourier map
*R*[*F*^2^ > 2σ(*F*^2^)] = 0.038	Hydrogen site location: inferred from neighbouring sites
*wR*(*F*^2^) = 0.111	H atoms treated by a mixture of independent and constrained refinement
*S* = 1.03	*w* = 1/[σ^2^(*F*_o_^2^) + (0.0527*P*)^2^ + 0.5702*P*] where *P* = (*F*_o_^2^ + 2*F*_c_^2^)/3
3548 reflections	(Δ/σ)_max_ = 0.001
189 parameters	Δρ_max_ = 0.33 e Å^−^^3^
0 restraints	Δρ_min_ = −0.26 e Å^−^^3^

### Special details

**Table:**

Experimental. The crystal was placed in the cold stream of an Oxford Cryosystems Cobra open-flow nitrogen cryostat (Cosier & Glazer, 1986) operating at 100.0 (1) K.
Geometry. All s.u.'s (except the s.u. in the dihedral angle between two l.s. planes) are estimated using the full covariance matrix. The cell s.u.'s are taken into account individually in the estimation of s.u.'s in distances, angles and torsion angles; correlations between s.u.'s in cell parameters are only used when they are defined by crystal symmetry. An approximate (isotropic) treatment of cell s.u.'s is used for estimating s.u.'s involving l.s. planes.
Refinement. Refinement of F^2^ against ALL reflections. The weighted R-factor wR and goodness of fit S are based on F^2^, conventional R-factors R are based on F, with F set to zero for negative F^2^. The threshold expression of F^2^ > 2σ(F^2^) is used only for calculating R-factors(gt) etc. and is not relevant to the choice of reflections for refinement. R-factors based on F^2^ are statistically about twice as large as those based on F, and R- factors based on ALL data will be even larger.

### Fractional atomic coordinates and isotropic or equivalent isotropic displacement parameters (Å^2^)

**Table:**

	*x*	*y*	*z*	*U*_iso_*/*U*_eq_	
Cl1	0.12582 (5)	0.88201 (4)	−0.28298 (2)	0.02899 (12)	
O1	0.20080 (17)	0.01744 (10)	0.13325 (8)	0.0279 (3)	
O2	0.37683 (16)	0.09144 (10)	0.27923 (7)	0.0273 (3)	
O3	0.44612 (16)	0.29081 (10)	0.29829 (7)	0.0267 (3)	
H1O3	0.5054	0.2615	0.3536	0.040*	
O4	0.38530 (16)	0.70448 (10)	0.06318 (7)	0.0259 (2)	
N1	0.26920 (16)	0.49306 (11)	0.01711 (8)	0.0191 (2)	
C1	0.3279 (2)	0.74509 (13)	−0.01393 (9)	0.0195 (3)	
C2	0.3424 (2)	0.86975 (14)	−0.03574 (10)	0.0217 (3)	
H2A	0.3936	0.9254	0.0059	0.026*	
C3	0.2821 (2)	0.91040 (14)	−0.11739 (10)	0.0218 (3)	
H3A	0.2931	0.9929	−0.1308	0.026*	
C4	0.20390 (19)	0.82731 (14)	−0.18053 (9)	0.0203 (3)	
C5	0.18541 (19)	0.70552 (14)	−0.16270 (9)	0.0194 (3)	
H5A	0.1326	0.6517	−0.2053	0.023*	
C6	0.24713 (18)	0.66229 (13)	−0.07925 (9)	0.0173 (3)	
C7	0.22361 (19)	0.53618 (13)	−0.06027 (9)	0.0190 (3)	
H7A	0.1746	0.4832	−0.1040	0.023*	
C8	0.24807 (19)	0.37125 (13)	0.04491 (9)	0.0187 (3)	
C9	0.1617 (2)	0.27914 (13)	−0.00918 (9)	0.0208 (3)	
H9A	0.1156	0.2968	−0.0660	0.025*	
C10	0.1456 (2)	0.16216 (14)	0.02226 (10)	0.0222 (3)	
H10A	0.0870	0.1013	−0.0134	0.027*	
C11	0.2162 (2)	0.13432 (13)	0.10694 (10)	0.0209 (3)	
C12	0.30148 (19)	0.22675 (13)	0.16180 (9)	0.0194 (3)	
C13	0.31643 (19)	0.34519 (13)	0.12975 (9)	0.0186 (3)	
H13A	0.3725	0.4068	0.1654	0.022*	
C14	0.3777 (2)	0.19733 (14)	0.25151 (10)	0.0217 (3)	
H1N1	0.322 (3)	0.5468 (19)	0.0518 (13)	0.029 (5)*	
H1O1	0.268 (3)	0.024 (2)	0.1903 (16)	0.052 (7)*	

### Atomic displacement parameters (Å^2^)

**Table:**

	*U*^11^	*U*^22^	*U*^33^	*U*^12^	*U*^13^	*U*^23^
Cl1	0.02706 (19)	0.0374 (2)	0.02110 (19)	0.00013 (15)	−0.00132 (13)	0.00998 (15)
O1	0.0366 (6)	0.0206 (5)	0.0258 (6)	−0.0036 (4)	0.0025 (5)	0.0019 (4)
O2	0.0324 (6)	0.0254 (6)	0.0238 (6)	−0.0010 (4)	0.0030 (5)	0.0057 (4)
O3	0.0361 (6)	0.0245 (5)	0.0177 (5)	0.0030 (4)	−0.0028 (4)	−0.0001 (4)
O4	0.0354 (6)	0.0273 (5)	0.0138 (5)	−0.0058 (4)	−0.0012 (4)	0.0009 (4)
N1	0.0199 (5)	0.0197 (6)	0.0175 (6)	−0.0016 (4)	0.0018 (4)	−0.0005 (5)
C1	0.0199 (6)	0.0228 (7)	0.0161 (6)	−0.0013 (5)	0.0036 (5)	−0.0005 (5)
C2	0.0230 (6)	0.0214 (7)	0.0209 (7)	−0.0046 (5)	0.0038 (5)	−0.0012 (5)
C3	0.0205 (6)	0.0216 (7)	0.0241 (7)	−0.0014 (5)	0.0058 (5)	0.0019 (5)
C4	0.0174 (6)	0.0252 (7)	0.0182 (7)	0.0017 (5)	0.0026 (5)	0.0047 (5)
C5	0.0169 (6)	0.0249 (7)	0.0165 (7)	−0.0011 (5)	0.0024 (5)	−0.0006 (5)
C6	0.0163 (6)	0.0198 (6)	0.0165 (6)	−0.0007 (5)	0.0043 (5)	−0.0001 (5)
C7	0.0176 (6)	0.0218 (6)	0.0177 (7)	−0.0003 (5)	0.0029 (5)	−0.0013 (5)
C8	0.0172 (6)	0.0204 (6)	0.0188 (7)	0.0019 (5)	0.0038 (5)	0.0011 (5)
C9	0.0209 (6)	0.0223 (7)	0.0184 (7)	0.0005 (5)	0.0002 (5)	−0.0010 (5)
C10	0.0217 (6)	0.0208 (7)	0.0234 (7)	−0.0011 (5)	0.0015 (5)	−0.0037 (5)
C11	0.0198 (6)	0.0213 (7)	0.0222 (7)	−0.0005 (5)	0.0045 (5)	−0.0004 (5)
C12	0.0175 (6)	0.0227 (7)	0.0186 (7)	0.0018 (5)	0.0043 (5)	−0.0005 (5)
C13	0.0178 (6)	0.0208 (6)	0.0174 (7)	0.0003 (5)	0.0032 (5)	−0.0018 (5)
C14	0.0208 (6)	0.0251 (7)	0.0197 (7)	0.0025 (5)	0.0047 (5)	0.0010 (5)

### Geometric parameters (Å, °)

**Table:**

Cl1—C4	1.7390 (15)	C4—C5	1.368 (2)
O1—C11	1.3502 (18)	C5—C6	1.4093 (19)
O1—H1O1	0.96 (3)	C5—H5A	0.9300
O2—C14	1.2355 (19)	C6—C7	1.423 (2)
O3—C14	1.3113 (19)	C7—H7A	0.9300
O3—H1O3	0.9687	C8—C13	1.3876 (19)
O4—C1	1.3051 (17)	C8—C9	1.402 (2)
N1—C7	1.3061 (19)	C9—C10	1.380 (2)
N1—C8	1.4143 (18)	C9—H9A	0.9300
N1—H1N1	0.85 (2)	C10—C11	1.393 (2)
C1—C2	1.410 (2)	C10—H10A	0.9300
C1—C6	1.4284 (19)	C11—C12	1.409 (2)
C2—C3	1.373 (2)	C12—C13	1.397 (2)
C2—H2A	0.9300	C12—C14	1.478 (2)
C3—C4	1.402 (2)	C13—H13A	0.9300
C3—H3A	0.9300		
			
C11—O1—H1O1	99.8 (15)	N1—C7—H7A	119.2
C14—O3—H1O3	109.3	C6—C7—H7A	119.2
C7—N1—C8	127.27 (13)	C13—C8—C9	120.25 (13)
C7—N1—H1N1	112.4 (14)	C13—C8—N1	117.00 (13)
C8—N1—H1N1	120.2 (14)	C9—C8—N1	122.76 (13)
O4—C1—C2	122.09 (13)	C10—C9—C8	119.69 (14)
O4—C1—C6	119.93 (13)	C10—C9—H9A	120.2
C2—C1—C6	117.99 (13)	C8—C9—H9A	120.2
C3—C2—C1	121.13 (14)	C9—C10—C11	120.64 (14)
C3—C2—H2A	119.4	C9—C10—H10A	119.7
C1—C2—H2A	119.4	C11—C10—H10A	119.7
C2—C3—C4	119.86 (14)	O1—C11—C10	117.85 (13)
C2—C3—H3A	120.1	O1—C11—C12	122.24 (14)
C4—C3—H3A	120.1	C10—C11—C12	119.91 (14)
C5—C4—C3	121.42 (14)	C13—C12—C11	119.18 (13)
C5—C4—Cl1	119.81 (12)	C13—C12—C14	120.76 (13)
C3—C4—Cl1	118.76 (11)	C11—C12—C14	120.04 (13)
C4—C5—C6	119.40 (13)	C8—C13—C12	120.31 (13)
C4—C5—H5A	120.3	C8—C13—H13A	119.8
C6—C5—H5A	120.3	C12—C13—H13A	119.8
C5—C6—C7	119.34 (13)	O2—C14—O3	123.20 (14)
C5—C6—C1	120.20 (13)	O2—C14—C12	121.53 (14)
C7—C6—C1	120.44 (13)	O3—C14—C12	115.27 (13)
N1—C7—C6	121.62 (13)		
			
O4—C1—C2—C3	179.64 (13)	C13—C8—C9—C10	−0.2 (2)
C6—C1—C2—C3	−0.5 (2)	N1—C8—C9—C10	−179.90 (13)
C1—C2—C3—C4	0.3 (2)	C8—C9—C10—C11	−0.8 (2)
C2—C3—C4—C5	0.2 (2)	C9—C10—C11—O1	−178.20 (13)
C2—C3—C4—Cl1	179.12 (11)	C9—C10—C11—C12	1.4 (2)
C3—C4—C5—C6	−0.4 (2)	O1—C11—C12—C13	178.57 (13)
Cl1—C4—C5—C6	−179.28 (10)	C10—C11—C12—C13	−1.0 (2)
C4—C5—C6—C7	178.48 (13)	O1—C11—C12—C14	−0.1 (2)
C4—C5—C6—C1	0.1 (2)	C10—C11—C12—C14	−179.59 (13)
O4—C1—C6—C5	−179.82 (13)	C9—C8—C13—C12	0.6 (2)
C2—C1—C6—C5	0.3 (2)	N1—C8—C13—C12	−179.70 (12)
O4—C1—C6—C7	1.8 (2)	C11—C12—C13—C8	0.0 (2)
C2—C1—C6—C7	−178.04 (13)	C14—C12—C13—C8	178.60 (13)
C8—N1—C7—C6	178.05 (13)	C13—C12—C14—O2	−175.13 (14)
C5—C6—C7—N1	−176.28 (13)	C11—C12—C14—O2	3.5 (2)
C1—C6—C7—N1	2.1 (2)	C13—C12—C14—O3	4.4 (2)
C7—N1—C8—C13	176.56 (13)	C11—C12—C14—O3	−176.96 (13)
C7—N1—C8—C9	−3.8 (2)		

### Hydrogen-bond geometry (Å, °)

**Table:**

*D*—H···*A*	*D*—H	H···*A*	*D*···*A*	*D*—H···*A*
O3—H1O3···O4^i^	0.97	1.56	2.5220 (16)	173.
N1—H1N1···O4	0.85 (2)	1.78 (2)	2.5217 (17)	144.2 (19)
O1—H1O1···O2	0.96 (2)	1.67 (2)	2.5901 (17)	158 (2)
C7—H7A···Cl1^ii^	0.93	2.81	3.6603 (15)	152.

Symmetry codes: (i) −*x*+1, *y*−1/2, −*z*+1/2; (ii) −*x*, *y*−1/2, −*z*−1/2.

## References

[bb1] Belaid, S., Djebbar, S., Benali-Baitich, O., Khan, M. & Bouet, G. (2006). *C. R. Chim.* **10**, 568–572.

[bb2] Bernstein, J., Davis, R. E., Shimoni, L. & Chang, N.-L. (1995). *Angew. Chem. Int. Ed. Engl.* **34**, 1555–1573.

[bb3] Bruker (2009). *APEX2*, *SAINT* and *SADABS* Bruker AXS Inc., Madison, Wisconsin, USA.

[bb4] Cosier, J. & Glazer, A. M. (1986). *J. Appl. Cryst.* **19**, 105–107.

[bb5] Karthikeyan, M. S., Prasad, D. J., Poojary, B., Bhat, K. S., Holla, B. S. & Kumari, N. S. (2006). *Bioorg. Med. Chem.* **14**, 7482–7489. 10.1016/j.bmc.2006.07.015 16879972

[bb7] Salih, I. & Hamdi, T. (2008). *J. Coord. Chem.* **62**, 456–464.

[bb8] Sheldrick, G. M. (2008). *Acta Cryst.* A**64**, 112–122. 10.1107/S0108767307043930 18156677

[bb9] Spek, A. L. (2009). *Acta Cryst.* D**65**, 148–155. 10.1107/S090744490804362X PMC2631630 19171970

[bb6] Youssef, N. S., El-Zahany, E. A., Barsoum, B. N. & El-Seidy, A. M. A. (2009). *Transition Met. Chem.* **34**, 905–914.

